# Investigating the Potential Use of Date Kernel Ash (DKA) as a Partial Cement Replacement in Concrete

**DOI:** 10.3390/ma15248866

**Published:** 2022-12-12

**Authors:** Muneer K. Saeed, Muhammad K. Rahman, Mohammed Alfawzan, Shameer Basha, Hany A. Dahish

**Affiliations:** 1 Department of Civil Engineering, College of Engineering, Qassim University, Unaizah 56452, Saudi Arabia; 2 Interdisciplinary Research Center for Construction and Building Materials, King Fahd University of Petroleum & Minerals, Dhahran 34462, Saudi Arabia; 3 Department of Mechanical Engineering, College of Engineering, Qassim University, Unaizah 56452, Saudi Arabia; 4 Civil Engineering Department, Faculty of Engineering, Fayoum University, Fayoum 63511, Egypt

**Keywords:** date kernel ash (DKA), cement replacement, fly ash, natural pozzolan, heat of hydration, thermal conductivity

## Abstract

The palm and date sector is one of the most important sectors in Saudi Arabia. The total number of fertile palm trees in Saudi Arabia is about 31 million. In the production of pitted dates, date molasses, date paste, and date confectionery, a considerable number of date kernels are usually discarded as waste. This study reports experimental investigations conducted to evaluate the potential of waste date kernel ash (DKA), obtained by the calcination of date pits at 800 °C, as a partial cement replacement in concrete. DKA has low silica oxide and does not qualify as a pozzolanic material. The effect of DKA partially replacing the cement and acting as a filler material in concrete was investigated, and its properties were compared with two pozzolanic materials, fly ash (FA) and natural pozzolan (NP). Twelve concrete mixes in which cement was replaced with different proportions of calcined DKA (5%, 10%, 15%, 20%, and 30%), NP (10%, 20%, and 30%), and FA (10%, 20%, and 30%) were investigated in the experimental program. The properties of DKA, FA, and NP concrete mixes were evaluated in fresh and hardened states, including the heat of hydration, mechanical characteristics, and thermal properties. The results show that replacing cement with 5% date kernel ash increases the compressive strength by 0.42%, 3.2%, and 2.5% at 3, 7, and 28 days, respectively, while the 28-day compressive strength decreases by 2.4%, 5.4%, 16.3%, and 26.69% when the cement is replaced with 10%, 15%, 20%, and 30% DKA, respectively. Date kernel ash concrete mixes with 10%, 20%, and 30% replacement levels demonstrated higher compressive and tensile strengths and lower thermal conductivity, density, and workability when compared to natural pozzolan and fly ash. DKA is a promising partial cement replacement material; nevertheless, additional research is required to assess the durability of DKA in concrete.

## 1. Introduction

**Figure 1:**
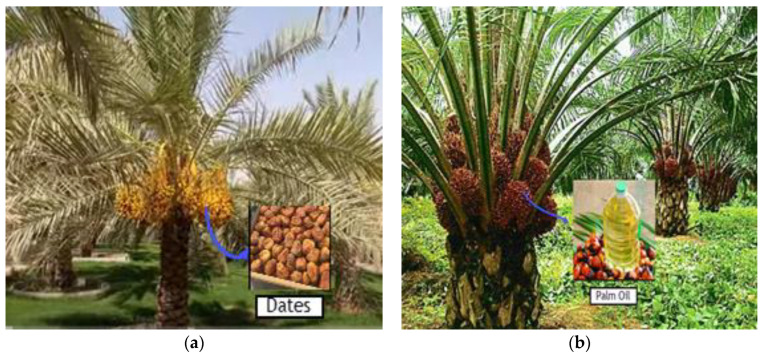
The two main types of palm trees: (**a**) date palm and (**b**) oil palm.

The date palm and oil palm are the two most widespread palm species. The date palm is most common in Middle Eastern countries, such as Saudi Arabia, Egypt, and the Emirates, as well as in the United States (California) [1], while the oil palm is mainly found in Malaysia and Indonesia [2]. Dates contain high concentrations of proteins, vitamins, and minerals [3], while oil palm was the main source of 35% of vegetable oil worldwide in 2019 [4]. Figure 1 shows date and oil palm trees.

**Figure 2:**
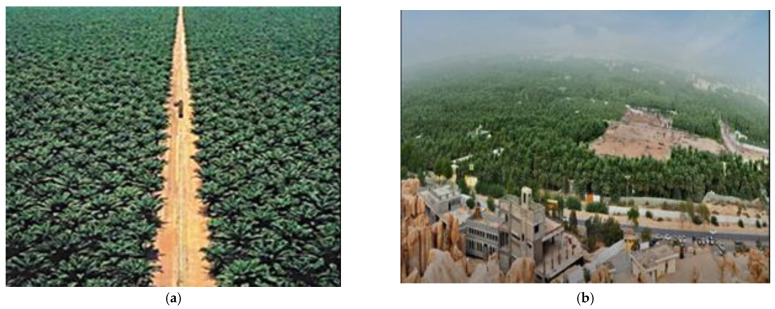
The largest palm tree farm and self-contained oasis in the world. (**a**) Date palm farm in Qassim, KSA; (**b**) largest self-contained oasis in the world.

The production of dates in Saudi Arabia accounts for 17% of the total worldwide output, making it one of the world’s largest date-producing countries. Saudi Arabia produces around 1.5 million tons of dates annually and has approximately 31 million palms [5]. According to the Guinness Book of Records for General Information and Standard Achievement, the largest date palm farm in the world, with 200,000 palm trees, is located in the southeastern region of Qassim in Saudi Arabia, and the largest enclosed oasis in the world is Al-Ahsa Oasis in southeastern Saudi Arabia, with 2.5 million palms [5]. Figure 2 shows a section of the largest palm tree farm in the Qassim region and Al-Ahsa Oasis. A portion of the dates harvested from palm trees are utilized to make a variety of products, such as date molasses, date jam, date paste, date vinegar, and date liquid sugar. A considerable quantity of date kernels is obtained from the production of date products and disposed of in landfills. Raw date kernel powder has been reported to be used in several applications, including food, medicines, cosmetics, nutrition, and coffee substitute, and is a promising source of oil [6,7]. The utilization of raw date kernel powder as a retarder for concrete was evaluated by the authors in a recent work [8]. Research on the use of date kernel ash as a partial cement replacement in concrete has been rarely reported in the literature. Al salami et al. [9] investigated the possibility of producing high-performance concrete by replacing coarse aggregates, fine aggregates, and cement with shells of palm kernel, powder palm kernels, and calcined kernel ash, respectively. They reported that the compressive strength of concrete with palm kernel ash decreased by about 28–40% with various replacement percentages between 5 and 20%. Alkutti [10] studied the potential of replacing cement with palm ash produced from burning dead palm fronds. They concluded that the optimal level of cement replacement with palm ash was 10% to enhance compressive strength at 360 days compared to normal concrete. The replacement of cement with date palm leaf ash (DPLA) at various replacement ratios (1%, 3%, 5%, 10%, and 15%) was investigated by Mehdi et al. [11]. They found that the optimal DPLA replacement ratios for compressive and flexural strengths are 3% and 5%, respectively. The environmental effect of replacing cement with palm ash in mortar was investigated by Nawaf [12]. The findings of his study show that when date ash is used as a cement replacement in mortar, there is no environmental risk to human health.

The utilization of palm oil fuel ash has, however, been extensively investigated and reported in the literature [13,14,15,16]. Studies on palm oil kernel ash (POFA) include the fresh and hardened properties of concrete when POFA replaced cement at percentages ranging from 5% to 70% [17,18]. The chemical composition of POFA includes mainly (59–65%) SiO_2_ and (4.8–8.2%) CaO [19,20,21,22]. This is significantly higher than the silica content in date kernel ash. The microstructure of POFA was investigated in [23,24], which shows that POKA has angular and irregular particles, with a sizable fraction showing cellular textures. The durability of POFA concrete has been investigated [25,26,27], and it was found that the addition of POFA to concrete improved the acid and sulfate resistance and reduced the dry shrinkage and surface water absorption of the concrete without affecting the final compressive strength. Palm oil clinker powder is recommended as a replacement for up to 40% of cement in masonry mortar due to its lower cost and lower environmental impact [28]. The replacement of cement with 40% nano-palm oil clinker powder leads to an 86.4% reduction in the compressive strength of concrete at 28 days with semi-leveled aggregate [29]. The potential for coarse aggregate replacement with oil palm shells (OPSs), also a bio-waste derived from the palm oil industry, has been investigated. It has been shown that OPSs can replace up to 75% of coarse aggregates at a 1:2:4 mix ratio and up to 50% at a 1:3:6 mix ratio [30]. Many studies have been conducted to develop lightweight concrete (LWC) with oil palm kernel shells as an aggregate [31,32,33,34,35].

Generally, mineral admixtures, such as natural pozzolan and fly ash, are used to partially replace Portland cement. Several studies in the literature have presented the use of natural pozzolan and fly ash as partial substitutes for cement in mortar and concrete [36,37,38,39]. In this study, waste date kernels were obtained from a date processing factory in Qassim region (KSA) and then separated, cleaned, crushed, thermally treated, and pulverized to generate date kernel ash (DKA). DKA was used as a partial replacement for cement in concrete mixes. The heat of hydration and thermal and mechanical properties of DKA, FA, and NP were compared.

## 2. Materials and Methods

### 2.1. Materials

**Figure 3:**
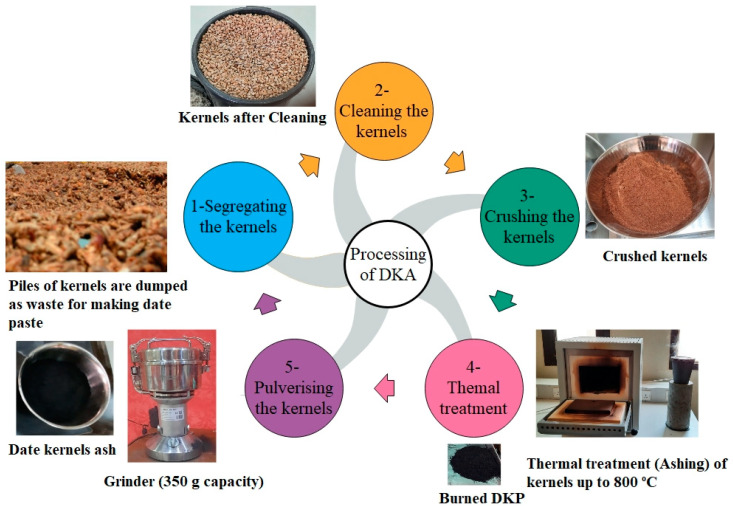
Process of making date kernel ash (DKA).

**Figure 4:**
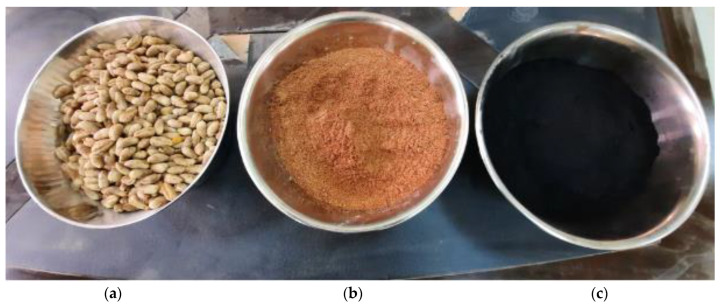
Physical appearance of date kernels. (**a**) Date kernels; (**b**) date kernel powder; (**c**) date kernel ash.

**Figure 5:**
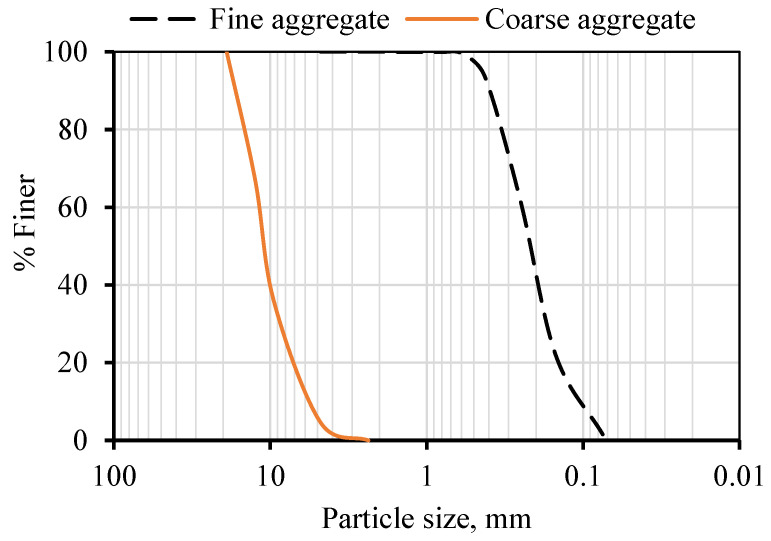
Particle size distributions of fine aggregate and coarse aggregate.

**Figure 6:**
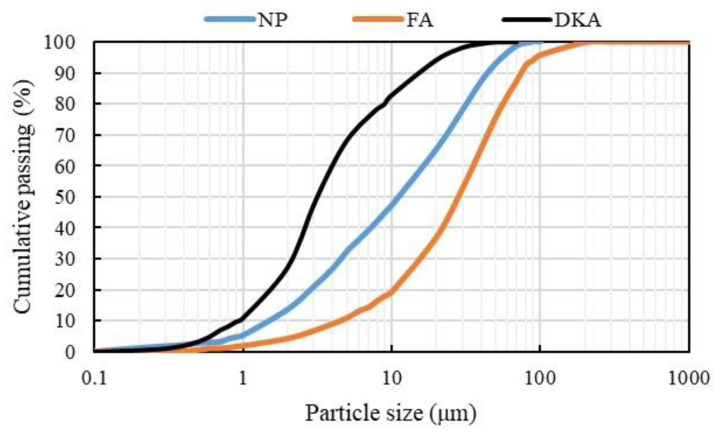
Particle size distributions of DKA, FA, and NP.

In this study, concrete mixtures were made using ordinary Portland cement (OPC) Type 1 from the Qassim cement plant, Buraiah, Saudi Arabia. Table 1 details the chemical compositions of OPC. The date kernels used in this study were obtained from a date factory in the Qassim region, Buraidah, Saudi Arabia and then sorted, cleaned, crushed, and thermally treated at different temperatures in a closed vessel oven until a temperature of 800 °C was reached. The thermal treatment was used to convert the date kernels from an organic to an ash state. After thermally treating the date kernels, they were ground to a fine powder with a particle size of less than 75 µm. Figure 3 shows the procedures for obtaining DKA. The date kernels, in their various forms (powder and ash), are shown in Figure 4. Type F fly ash with a specific gravity of 2.2 was used in this study. This type of fly ash is pozzolanic, which means it contains at least 70% silica, alumina, and ferrite oxide. The natural pozzolan was collected in the form of pellets from the western region of Saudi Arabia, which were then processed into powder in the lab using a grinder. The fineness of natural pozzolan after grinding ranged from 2000 kg/cm^2^ to 3000 kg/cm^2^. The fine aggregate used is locally available dune sand. It has a specific gravity of 2.6% and a water absorption of 0.3 %. The coarse aggregate is a limestone aggregate, with a specific weight of 2.6 and a water absorption of 1.4 %. The gradation of the fine and coarse aggregates is shown in Figure 5. Fluidum PC314 was used for concrete mixes as a liquid superplasticizer. It is brownish in color, and its specific gravity at 20 °C is 1.1. The particle size distributions of DKA, FA, and NP are shown in Figure 6. The physical properties, such as specific surface area, specific gravity, and water absorption, of DKA, FA, and NP are illustrated in Table 2. It is clear from Table 2 that the specific gravity of DKA is lower than that of FA and NP, while its water absorption is higher than that of FA and NP.

### 2.2. Microstructural Characterization of DKA

**Figure 7:**
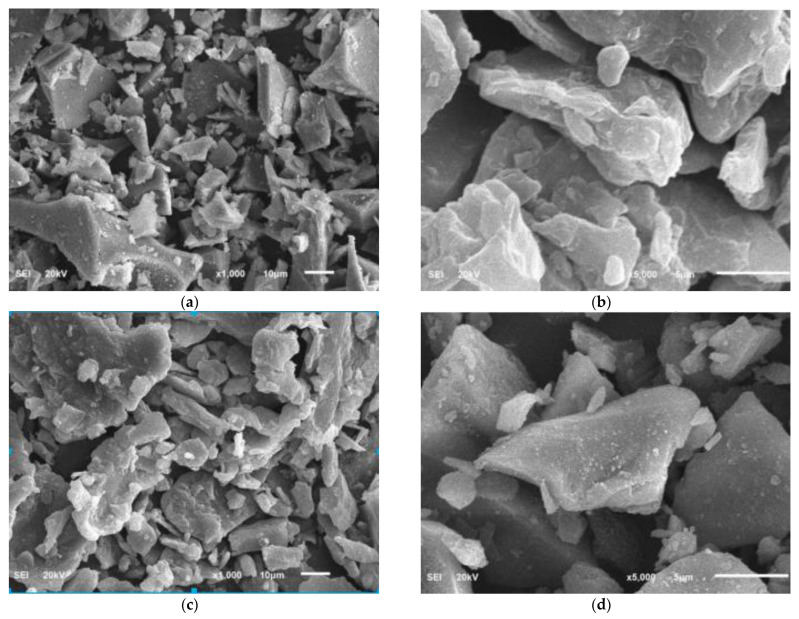

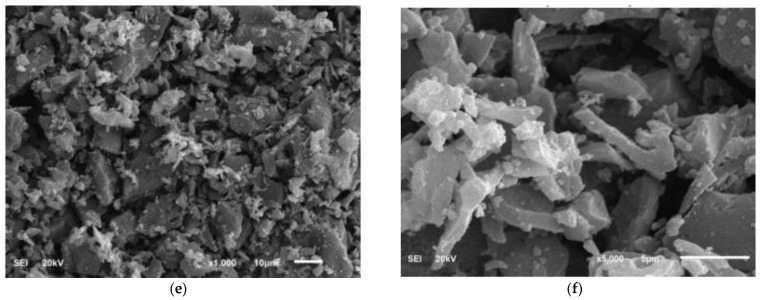
SEM image of natural pozzolan, raw kernel powder, and burnt kernel ash DKA. (**a**) Natural pozzolan (NP) at 10 µm; (**b**) natural pozzolan (NP) at 5 µm; (**c**) raw kernel (RK) powder at 10 µm; (**d**) raw kernel (RK) powder at 5 µm; (**e**) date kernel ash (DKA) at 10 µm; (**f**) date kernel ash (DKA) at 5 µm.

Figure 5 shows the morphology of the natural pozzolan, raw date kernel powder, and date kernel powder calcined at 800 °C; the images were obtained through JSM-5800LV (JEOL, Tokyo, Japan) scanning electron microscopy (SEM). The SEM of raw NP particles (Figure 7a,b) shows that the powder particles are irregular and angular with various sizes and shapes and sharp edges. The voids in the NP powder are evident in the figure. Elemental analysis shows the presence of silicon, carbon, iron, sodium, calcium, and magnesium in the powder. The SEM image of raw kernel powder (RKP) is shown in Figure 7c,d. The particle shapes and sizes vary significantly, with 3D and thin 2D particles with prominent voids. The SEM of burnt kernel ash DKA at magnifications of 10 µm and 5 µm are shown in Figure 7e,f. As observed in the figure, the shape of the powder particles is, to a great extent, irregular and angular. Particles are thin and oblong, and some of them are cuboidal in shape with sharp edges, while others are flaky. The elemental analysis indicates the presence of calcium, potassium, silica, magnesium, and other elements.

### 2.3. Proportions of Concrete Mixtures

In this study, a total of twelve different types of concrete mixtures were prepared. The reference mix (Mix 1) had an OPC content of 480 kg/m^3^ and a water–cement ratio of 0.38. In Mix Nos. 2 to 6, varying percentages of thermally treated date seed powder (DKA) were used in place of cement (5%, 10 %, 15%, 20%, and 30%). In Mix Nos. 7 to 9, fly ash was used in place of cement at various percentages (10%, 20%, and 30%). In Mix Nos. 10 to 12, cement was replaced with 10%, 20%, and 30% natural pozzolan. The ratio of water to binder was fixed for all mixtures at 0.38. Table 3 shows the mix constituents for the twelve concrete mixes.

### 2.4. Thermal Treatment of DKA

**Figure 8:**
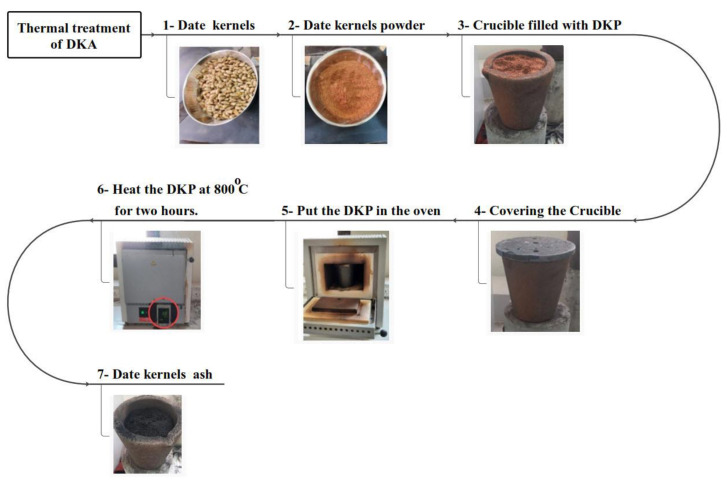
Thermal treatment of DKA.

The date kernels were cleaned and ground before being placed in a graphite crucible. The crucible has a height of 14 cm and a diameter of 14.5 cm and can withstand temperatures up to 1600 °C. The crucible was then heated to 800 °C within 2 h in a closed-vessel oven. The temperature was then maintained at 800 °C for 2 h before gradually cooling to 30 °C at a rate of 50 °C/h. Figure 8 illustrates the thermal treatment processes of DKA.

### 2.5. XRF of DKA, FA, and NP

**Figure 9:**
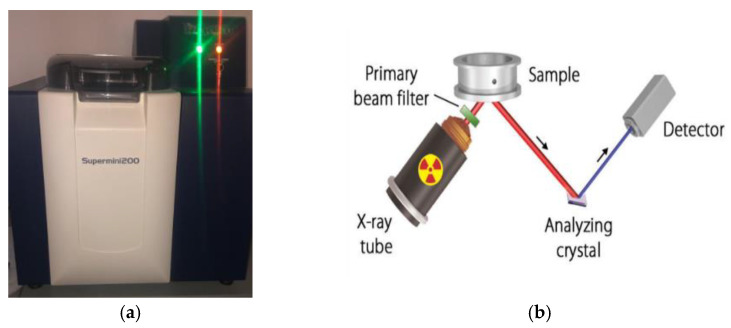
WDXRF analysis. (**a**) Supermini200 High-Power Benchtop Sequential WDXRF Spectrometer; (**b**) elemental analysis of specimens.

Rikagu’s supermini200 spectrometer (Ragaku Americas Corporation, The Woodlands, TX, USA) was used for the X-ray fluorescence experiment to determine the elemental compositions of DKA, NP, and FA (see Figure 9a). The samples are exposed to energized photons from an X-ray beam, which alters the atomic orbits of electrons and releases fluorescent photons, as shown in Figure 9b. The elements in the sample are then determined by evaluating the X-ray wavelengths released [40,41]. XRF analysis shows that the loss on ignition in natural DKA is very high, because it is an organic substance with high carbon and low percentages of chemical oxides. DKA thermally treated at 800 °C has larger amounts of silicon dioxide (SiO_2_) and calcium oxide (CaO), as shown by the XRF analysis (Table 4). As the treatment temperature increases, the percentages of chemical oxides or elements grow, while the residual organic materials decrease. Table 4 shows the WDXRF analysis of DKA in the natural state and after thermal treatment at 200 °C and 800 °C, as well as for NP and FA. The XRF of DKA calcined at 800 °C (designated as DKA) shows that it contains more CaO (33.4%) compared to FA and NP. Both FA and NP have a higher percentage of SiO_2_ at 52% and 55.8%, respectively. XRF shows that DKA cannot be classified as a pozzolanic material due to its low SiO_2_ content.

### 2.6. Heat of Hydration

**Figure 10:**
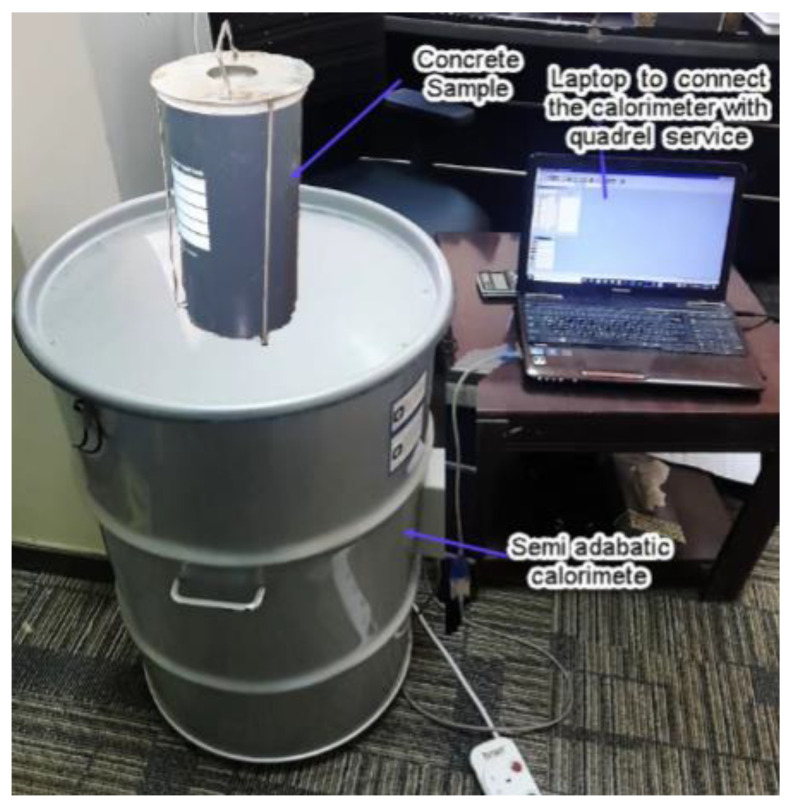
iQDrum for measuring heat and temperature generation in concrete.

The adiabatic heat of hydration of DKA and other materials was determined using the semi-adiabatic calorimeter iQDrum (Quadrel Inc., Pittsburgh, PA, USA)*,* as shown in Figure 10. There are two sensors in the semi-adiabatic calorimeter for measuring the heat of hydration temperature generated at the core of a 300 mm × 150 mm cylindrical concrete sample and the heat loss through the calorimeter’s highly insulated walls. A data logger in the calorimeter records the data from the sensors at 15 min intervals. The data are sent to the attached computer and then transmitted to Quadrel iService for analysis (Figure 10). By integrating the sensor readings, the adiabatic heat rise and temperature increment of the concrete mix are computed. The test is continued for either 7 or 14 days, depending on the type of concrete mixture being tested [42].

### 2.7. Thermal and Mechanical Properties of Concrete Mixtures

**Figure 11:**
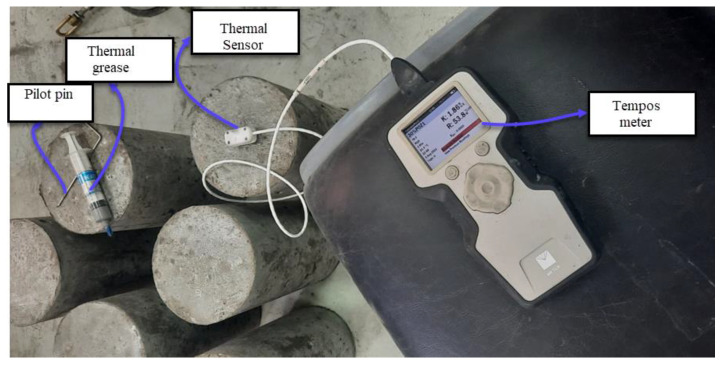
TEMPOS meter for thermal properties of concrete.

**Figure 12:**
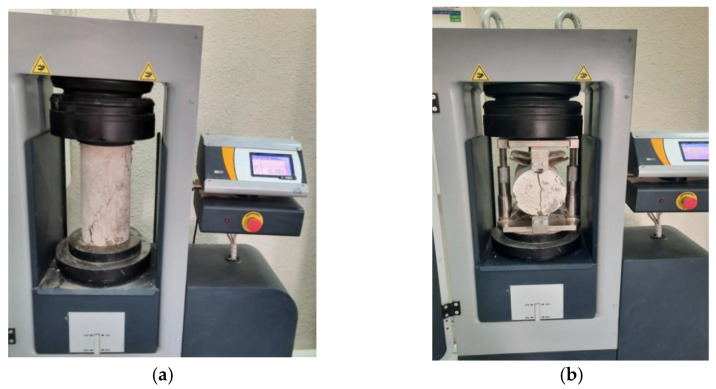
Measuring the mechanical properties of concrete. (**a**) Compressive strength; (**b**) Splitting tensile strength.

The thermal properties of DKA-, FA-, and NP-based concrete mixes were determined at 3, 7, and 28 days of age using the TEMPOS meter (METER Group, Inc., Pullman, WA, USA). Thermal conductivity, resistivity, and heat capacity were measured for the selected concrete mixtures. The thermal properties were measured with the meter using a heat pulse approach. A heating needle and a thermal sensor were inserted into the concrete, and then heat was applied for 60 s to the heating needle and, simultaneously, the concrete temperature was measured using the thermocouple. For the accuracy of measurements, good thermal contact needs to be ensured between the sensor and the concrete [43,44]. A pilot hole was drilled in the concrete cylinder and cleaned using compressed air, and then the hole was lubricated with grease and a thermal paste, which ensures good thermal contact. The sensors were then placed in the hole to measure the thermal properties (Figure 11). The mechanical properties of DKA, FA, and NP concrete mixes were determined following ASTM standards. The compressive strength of the concrete cylinder (150 mm × 300 mm) was determined as per ASTM C39 [45], and the split tensile strength of the cylinder was determined in accordance with ASTM C496 [46]. The compressive and splitting tensile strengths of the concrete mixes were determined at 3, 7, and 28 days. A 2000 kN capacity compression testing machine (CONTROLS Groups, Saronno, Italy) was used to determine the compressive and tensile strengths of the specimens, as shown in Figure 12.

### 2.8. Ultrasonic Pulse Velocity (UPV) of Concrete Mixes

**Figure 13:**
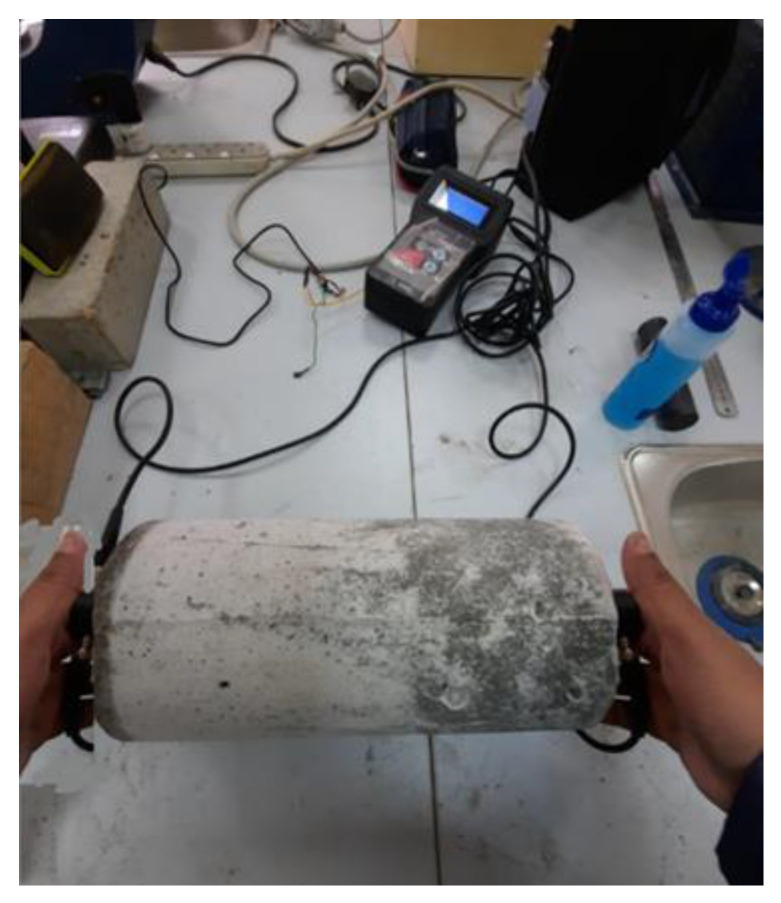
UPV of the concrete mixes.

The overall quality of the concrete and the identification of voids or cracks that may be present in the concrete matrix can be determined using the UPV method [47,48]. An ultrasonic pulse is generated and sent into the concrete sample, and the time taken by the pulse to pass through the sample is measured. Two transducers were placed on the two opposite faces of the cylindrical concrete specimen (150 mm × 300 mm), as shown in Figure 13. The UPV in the concrete cylinder sample was measured at 3, 7, and 28 days in both the transverse and longitudinal directions.

## 3. Results and Discussion

### 3.1. Workability and Density of Concrete Mixtures

**Figure 14:**
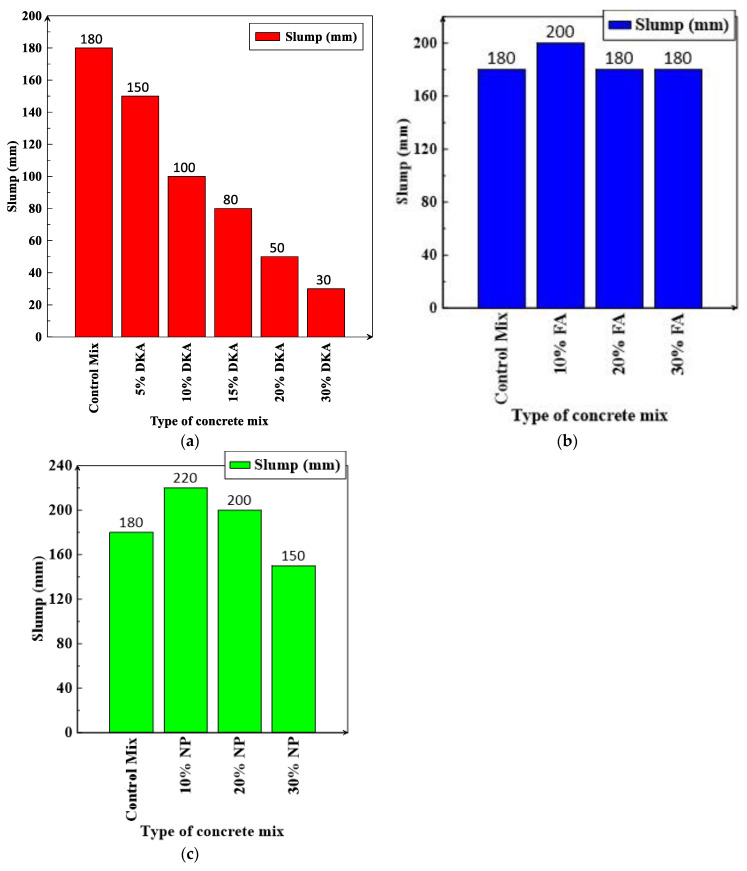
Slump of concrete mixes containing (**a**) DKA, (**b**) FA, and (**c**) NP.

Workability refers to how easily and consistently a freshly made concrete mix can be poured, compacted, and finished. The workability of concrete improves as the water–cement ratio increases and is generally measured by conducting slump tests on fresh concrete [49,50]. The slump values for the twelve concrete mixes were measured according to ASTM C134 and are shown in Figure 14a–c. As the percentage of DKA in the concrete mix increases, the slump and workability of the concrete mix decrease. When 5% of the cement is replaced by DKA, the slump value is 150 mm; however, when 30% of the cement is replaced by DKA, the value drops to 30 mm. In comparison to DKA concrete mixes, concrete mixes with various percentages of NP and FA have high slump values ranging from 200 mm to 180 mm for FA concrete mixes and 220 mm to 150 mm for NP concrete mixes. It is likely that the higher water absorption of DKA in comparison to FA and NP was the main reason for the decrease in the workability of concrete mixes with DKA. Conversely, the fly ash and natural pozzolan mixes had higher workability due to their lower water absorption rates compared to DKA, in addition to the lubricating effect that their spherical or spheroidal shape provides [51].

**Figure 15:**
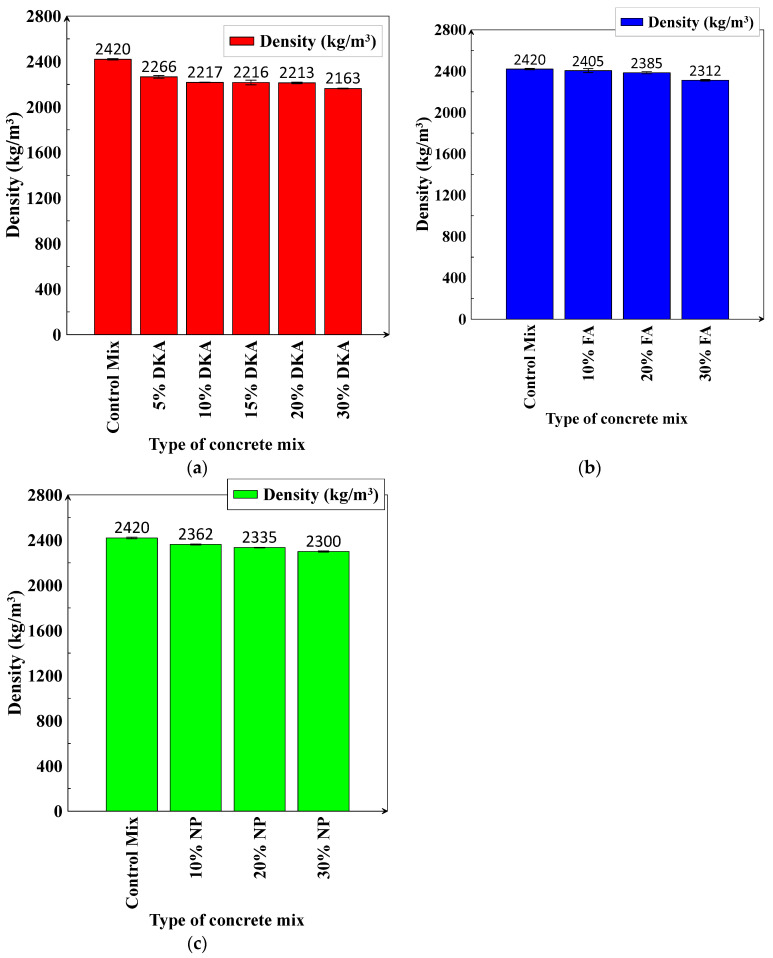
Density of concrete mixes containing (**a**) DKA, (**b**) FA, and (**c**) NP.

A reduction in the density of the concrete leads to a lightweight overall structure. When the density of the concrete is between 1361 kg/m^3^ and 1842 kg/m^3^, the concrete is classified as “light” [52]. The measured densities of fresh concrete for the 12 mixes investigated are shown in Figure 15. The density was measured based on the average values of weight/volume for three cylindrical specimens (15 cm × 30 cm). The densities of the concrete mixtures decrease noticeably when cement is replaced with various levels (10%, 20%, and 30%) of DKA. The replacement of cement with 10%, 20%, and 30% DKA decreased the density by 8.38%, 8.55%, and 10.61%, respectively, compared to the control mix (Figure 15a). The density of the concrete is not profoundly changed when cement is replaced with FA or NP, and the decrease in density is limited to a maximum of 5% when cement is replaced with 30% of FA or NP. The decrease in the density of concrete mixes containing DKA is presumed to be low due to its low specific gravity or density when compared to FA and NA, as indicated in Table 2. The 10.61% reduction in the concrete density caused by the replacement of cement with 30% DKA does not qualify it as lightweight concrete according to ACI standards. [52]. It is expected that increasing the replacement level of DKA to 50% or more will possibly result in a significant reduction in the density, at the cost of a decrease in compressive or tensile strength.

### 3.2. Heat of Hydration and Temperature Rise in Concrete Mixtures

**Figure 16:**
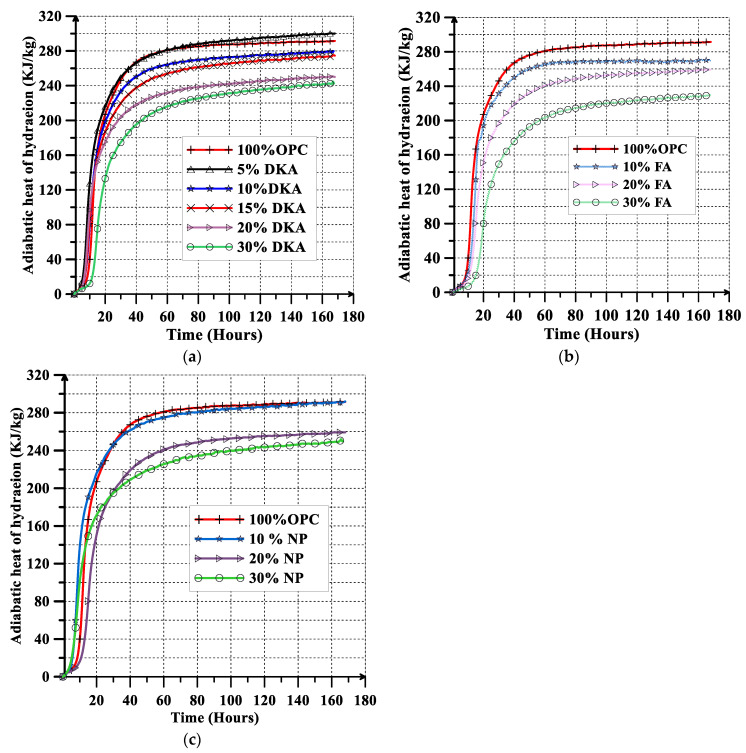
Adiabatic heat of hydration rise versus time for (**a**) DKA, (**b**) FA, and (**c**) NP.

The evolution of the adiabatic heat of hydration over time for the investigated concrete mixtures (12 mixes) is shown in Figure 16a–c. Replacing cement with 5% DKA leads to a slight increase in the adiabatic heat of hydration of about 3% compared to the control mix. Increasing the replacement level beyond 10% DKA results in a significant reduction in the heat of hydration. Concrete mixes with 10% and 20% DKA generate less heat of hydration than mixes with 10% and 20% FA, respectively. Replacing cement with 30% FA resulted in a greater reduction in heat of hydration than 30% DKA or 30% NP. The FA and NP mixes have a decreased heat of hydration due to a slower hydration reaction, which delays the time to reach the peak temperature, coupled with a decrease in the amplitude of the peak temperature. DKA on the other hand, in spite of having a higher surface area, reduces the heat of hydration, as its mechanism of action is as a filler because it is a non-pozzolanic material. At 5%, hydration might be accelerated due to the DKA filler providing nucleation sites for promoting the hydration reaction. When DKA replaces cement at 10–30%, the decrease in the cement content and higher number of filler particles filling the pores results in a significant reduction in the heat of hydration.

**Figure 17:**
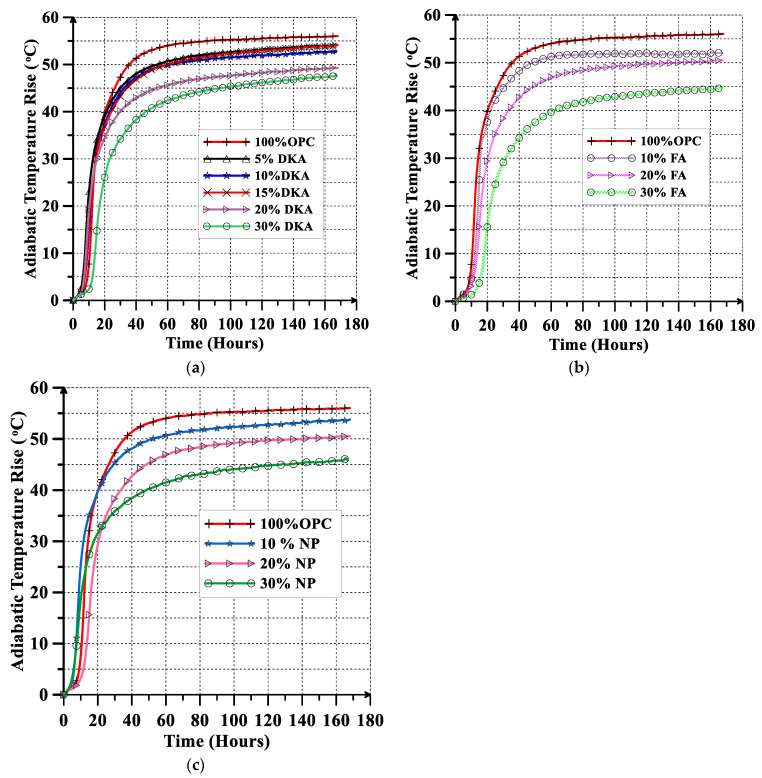
Adiabatic temperature rise versus time: (**a**) DKA, (**b**) FA, and (**c**) NP.

The heat of hydration obtained from semi-adiabatic calorimetry can be converted to the temperature rise under adiabatic conditions using the following equation [53,54]:

(1) $$\mathrm{ATR}=\frac{QW_{\mathrm{cm}}}{\gamma c}$$

where ATR is the adiabatic temperature rise (°C), Q is the heat of hydration (KJ/kg), W_cm_ is the weight of cementitious material per cubic meter (kg/m^3^), γ is the unit weight of concrete (kg/m^3^), and c is the specific heat capacity (KJ/m^3^.°C). The adiabatic temperature rises for the investigated concrete mixes are shown in Figure 17a–c. The control mix with 100% OPC has a high (55 °C) adiabatic temperature rise, which decreases as cement is replaced with various percentages of DKA, FA, and NP, as shown in Figure 15a–c. No significant difference was found between DKA, NP, and FA in terms of a reduction in the temperature rise at a replacement level of 10%. At a replacement level of 20%, DKA has a greater effect on lowering the temperature rise than either NP or FA. The mix with 30% FA has the greatest impact on reducing the temperature rise, as shown in Figure 17a–c.

**Figure 18:**
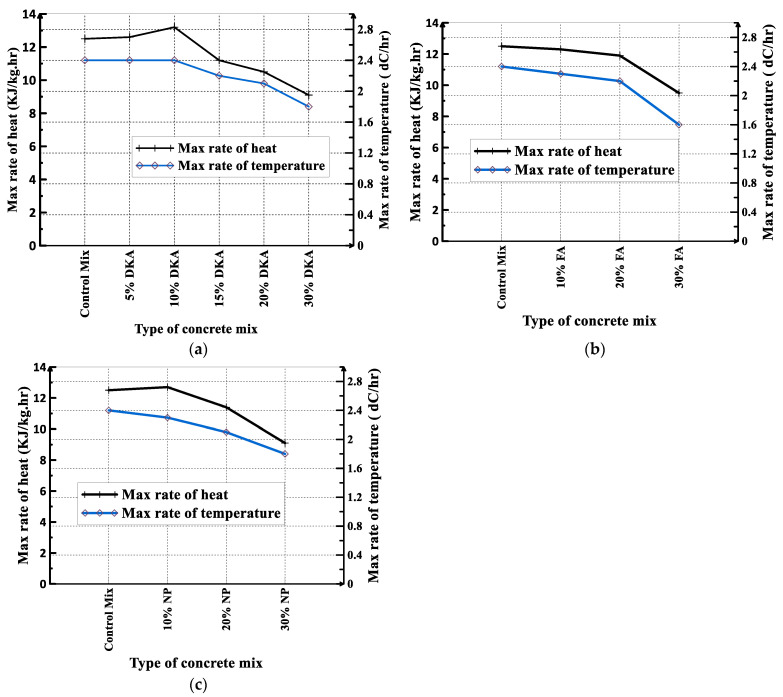
Rate of heat and temperature rise for concrete mixes containing (**a**) DKA, (**b**) FA, and (**c**) NP.

The maximum rate of change in the heat of hydration and the rate of the temperature rise measured for the twelve concrete mixes are shown in Figure 18a–c. The mix with a 10% DKA replacement has the highest hydration rate, 13.2 KJ/kg.hr, while the mix with a 30% DKA replacement has the lowest hydration rate, 9.1 KJ/kg.hr, as shown in Figure 18a. The temperature rise rates for the control mixture and the mixtures with 5% and 10% DKA were 2.4 dC/h. The mixture containing 30% FA had the lowest temperature rise rate, 1.6 dC/h. The rate of the temperature rise gradually decreases, as seen in Figure 18a–c, when the percentage of replacement with DKA, NP, and FA increases from 10% to 30%.

### 3.3. Thermal Conductivity, Resistivity, and Specific Heat

**Figure 19:**
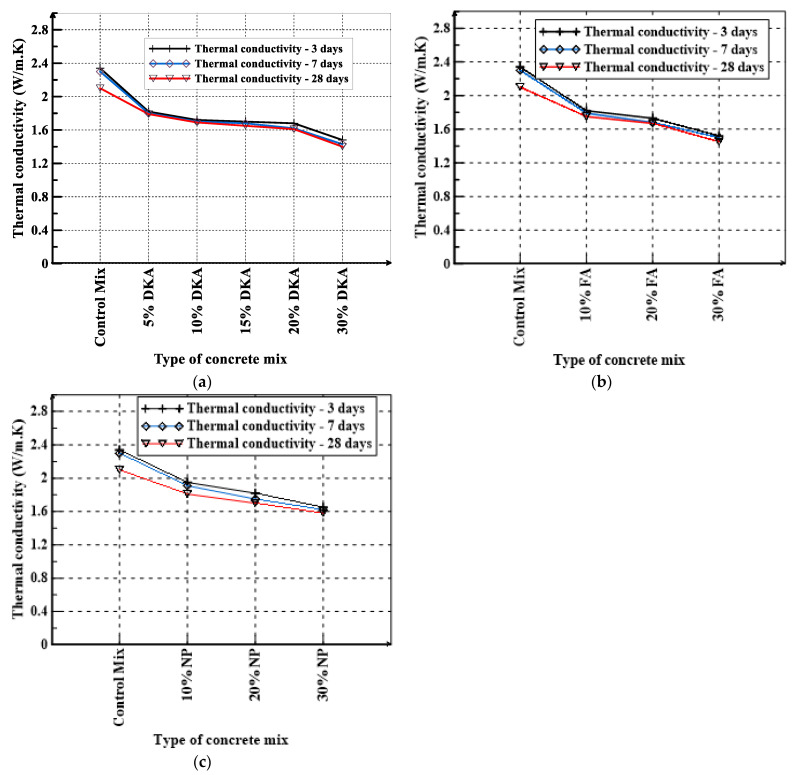
Thermal conductivity of (**a**) DKA, (**b**) FA, and (**c**) NP concrete mixes.

**Figure 20:**
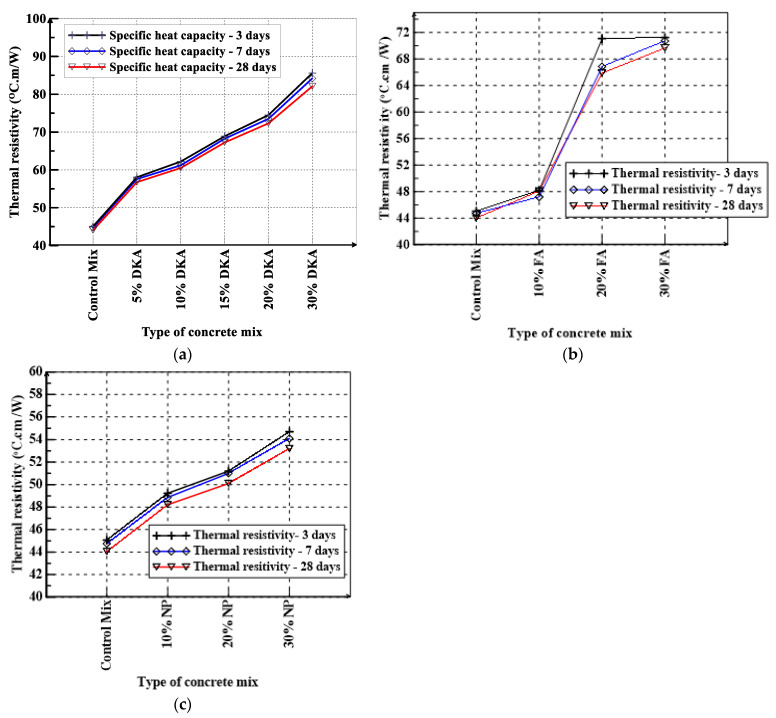
Thermal resistivity of (**a**) DKA, (**b**) FA, and (**c**) NP concrete mixes.

The TEMPOS meter was used to determine the thermal conductivity, resistivity, and specific heat capacity of concrete mixtures at 3, 7, and 28 days. The thermal conductivity, resistivity, and specific heat capacity of concrete mixtures investigated in this study are shown in Figure 18, Figure 19 and Figure 20. As can be seen in Figure 19a, DKA has a significant effect on lowering the thermal conductivity of concrete while increasing its thermal resistivity. The control mix has the highest thermal conductivity of 2.34 W/m.K, whereas the mix with 30% DKA has the lowest thermal conductivity of 1.4 W/m.K. The thermal conductivity drops noticeably by 40.2% when the cement is replaced with 30% DKA. The effect of NP on reducing thermal conductivity is less than that of DKA and FA, as can be seen in Figure 18c. Figure 19 shows that the thermal conductivities of the DKA, FA, and NP concrete mixes at 28 days are lower than the thermal conductivities measured at 3 days by 3% to 10%. In general, the thermal conductivity of the concrete mixes decreases with an increase in the replacement levels of DKA, NP, and FA. The thermal conductivity of the concrete mixes decreases because the density of the concrete with DKA is significantly lower than those of the NP- and FA-based concrete mixes. A lower density means increased porosity and less contact with the solid phases in the matrix, which reduced the thermal conductivity of the DKA mixes. FA and NP also have almost one-third lower specific gravity compared to OPC, which results in lower density, and it can be seen in Figure 19 that their thermal conductivities decrease as the FA and NP contents increase in the concrete mix. Both FA and NP concrete mixes exhibit lower thermal conductivity compared to the OPC control mix.

**Figure 21:**
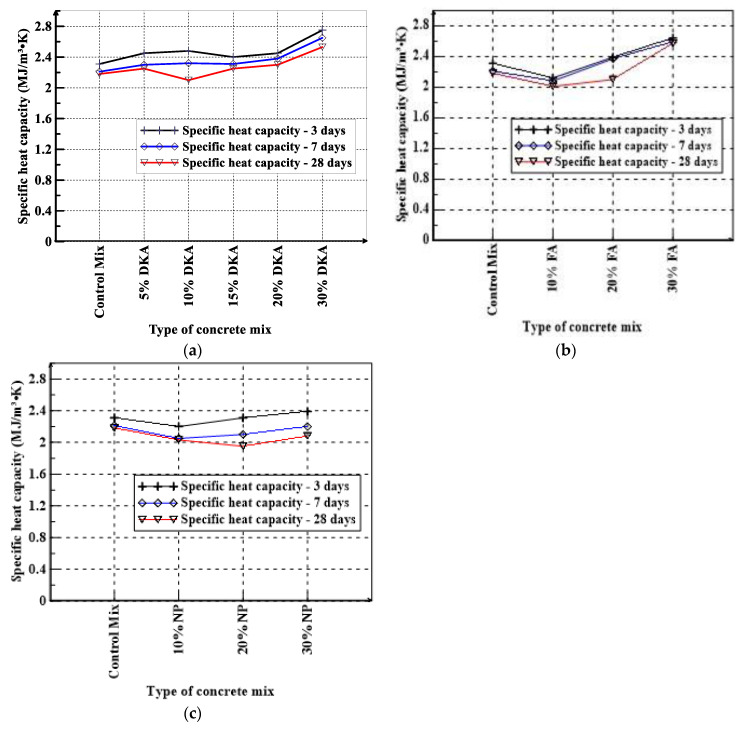
Specific heat of (**a**) DKA, (**b**) FA, and (**c**) NP concrete mixes.

The maximum thermal resistance was measured for the concrete mix with 30% DKA at the age of 28 days with a value of 85.6 °C.cm/W. Compared to the FA and NP mixes, the thermal resistance of the DKA mixes is higher, as can be seen in Figure 20a–c. NP and FA increase the thermal resistance of the concrete, but not to the same extent as DKA. The thermal resistance of DKA concrete with a 30% replacement of cement increases the thermal resistivity by almost 90% compared to the control OPC mix, whereas the FA and NP concrete increases are about 60% and 20%, respectively. The increase in thermal resistivity can be attributed to higher porosity and air voids in the DKA mixes. Although NP and FA concrete mixes at a 30% replacement of cement have similar densities, the thermal resistance of FA concrete is significantly higher. FA may have more porous particles compared to NP. The specific heat capacity is a measure of the amount of heat energy absorbed or released by a material as a function of the temperature difference and mass [55]. The measurement results of specific heat capacity for the twelve concrete mixes at 3, 7, and 28 days of age are shown in Figure 21a–c. The control mix has a volumetric specific heat of 2.31, 2.2, and 2.18 MJ/m^3^ K at the ages of 3, 7, and 28 days, respectively. The mixture with 30% DKA had the highest volumetric specific heat (2.75 MJ/m^3^ K).

When cement was replaced with 30% FA, the specific heat capacity increased by 17.88%, 17.19%, and 14.28% at 3, 7, and 28 days, respectively (Figure 20), However, 30% NP resulted in a decrease in the specific heat capacity to 29.48%, 29.56%, and 24.76% at 3, 7, and 28 days, respectively. The specific heat capacity of the DKA mixes is reduced significantly with age, as more free water gets physically bound to the gel products. The specific heat capacity for 30% DKA concrete is significantly higher than the 20% DKA concrete, as a higher replacement of cement by DKA as a filler material decreases the hydration reaction, so there is less physically bound water in the gel, and the specific heat capacity increases. A similar observation can be made with respect to the FA-based concrete mixes; however, NP concrete mixes have reduced specific heat at 7 and 28 days for 10, 20, and 30% cement replacements compared to the OPC mix. NP possibly has more water physically bound to the gel.

### 3.4. Compressive and Tensile Strengths of Concrete Mixtures

**Figure 22:**
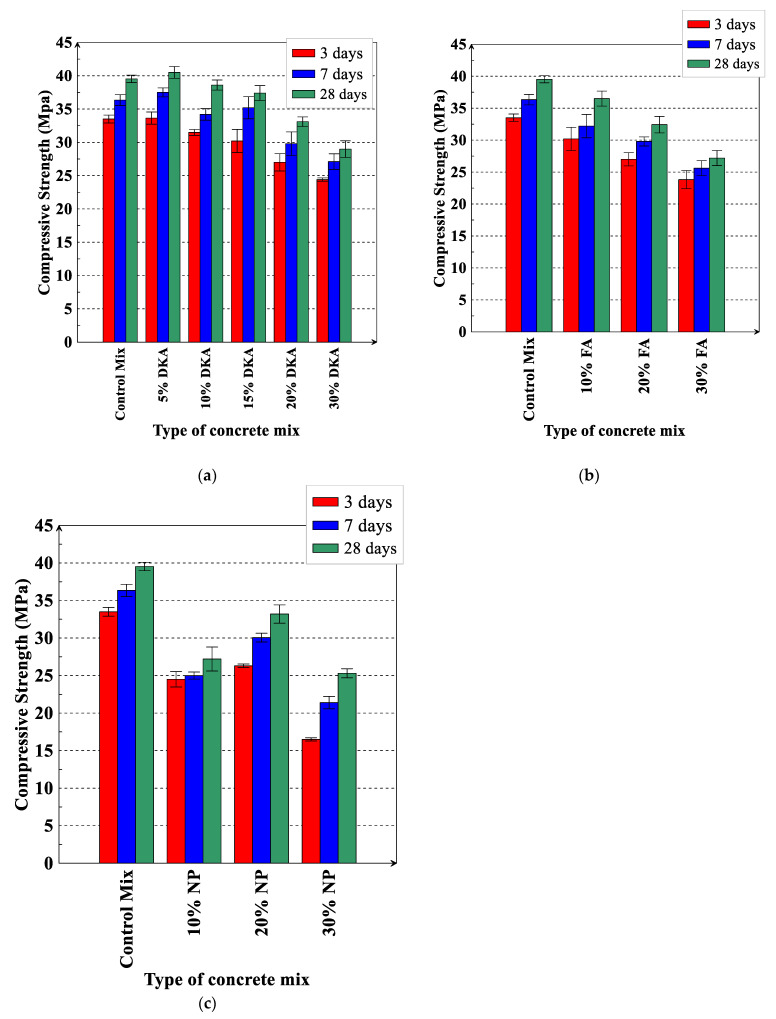
Compressive strength of concrete mixtures for (**a**) DKA, (**b**) FA, and (**c**) NP. Error bars represent one standard deviation from the average of three specimens.

The compressive and splitting tensile strengths of the twelve concrete mixtures were measured at 3, 7, and 28 days. Figure 22a–c depict the compressive strength results of the twelve concrete mixtures. The measured compressive strength of the control mix was 33.5 MPa, 36.43 MPa, and 39.53 MPa at 3, 7, and 28 days, respectively. The compressive strength of the mix with 5% DKA is 0.42%, 3.09%, and 2.4% higher than that of the control mix at the ages of 3, 7, and 28 days, respectively. This indicates that 5% DKA has a positive effect on the compressive strength of concrete. The higher compressive strength can be attributed to the filler effect of DKA, in which DKA particles provide nucleation sites for C-S-H, accelerating the hydration of the cement particles.

**Figure 23:**
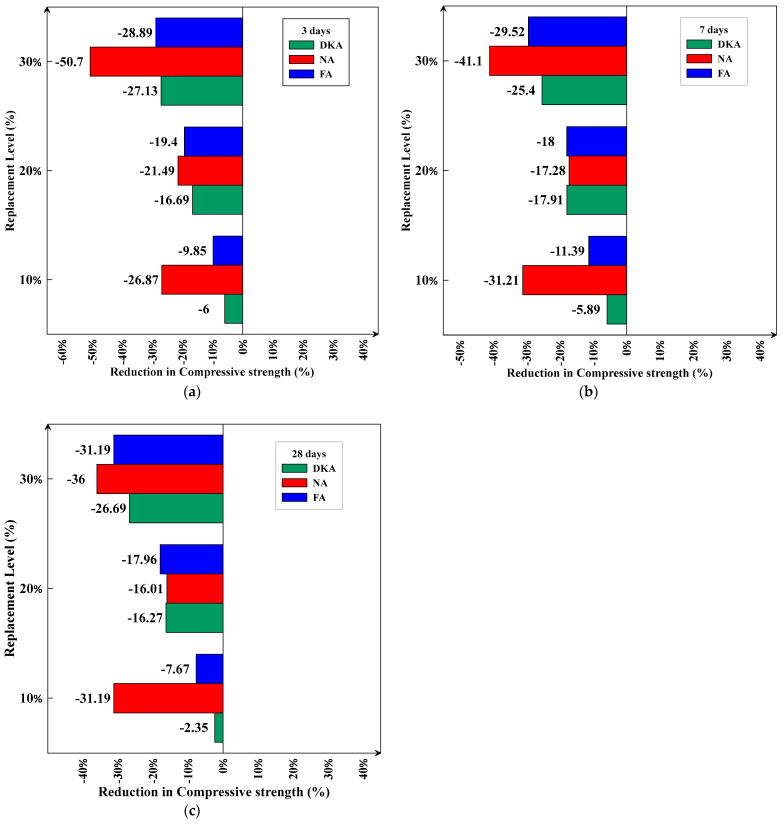
Reduction in compressive strength %. (**a**) DKA, (**b**) FA, and (**c**) NP.

The replacement of cement with 10% DKA led to reductions in compressive strength of 6%, 5.89%, and 2.35% at 3, 7, and 28 days of age, respectively, whereas 10% NP and 10% FA reduced the compressive strength by 26.87%, 31.21%, and 31.19 and 9.85%, 11.39%, and 7.67% at the same ages, respectively. In general, the replacement of cement with 10%, 20%, and 30% DKA, NP, and FA resulted in a decrease in compressive strength of varying percentages, as shown in Figure 23a–c. The mix with 10% DKA has the lowest drop in compressive strength (2.35% at 28 days), while the mix with 30% NP has the highest reduction (40% at 3 days). At 10%, 20%, and 30% replacements, it is interesting to notice that DKA has a higher compressive strength than NP and FA. Acting as a filler in the pores, the non-pozzolanic DKA particles possibly stimulate C-S-H nucleation. NP and FA, being pozzolanic, may not provide the same effect.

**Figure 24:**
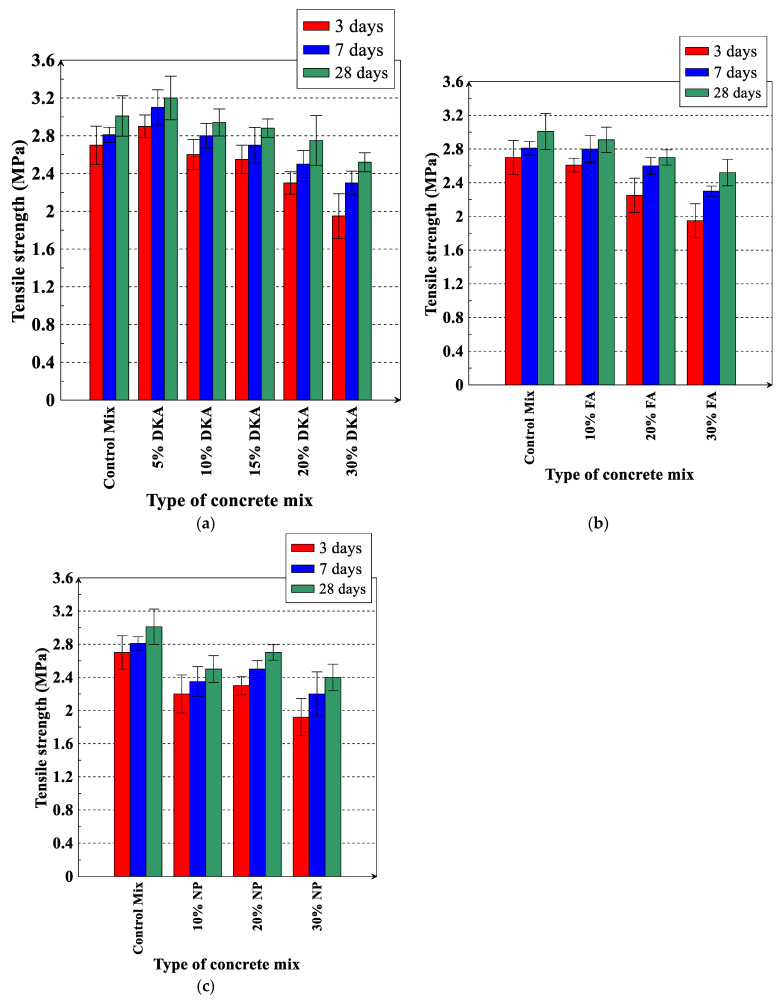
Tensile strength of concrete mixtures for (**a**) DKA, (**b**) FA, and (**c**) NP. Error bars represent one standard deviation from the average of three specimens.

The tensile strength of concrete mixtures at 3, 7, and 28 days of age was measured and is shown in Figure 24a–c. It can be seen in Figure 21 that the effects of DKA, NP, and FA on the tensile strength of concrete are approximately the same as their effects on the compressive strength of concrete. The tensile strength of the mix containing 5% DKA is 6.89%, 9.35%, and 5.93% higher than the control mix at 3, 7, and 28 days of age, respectively. At replacement percentages of 10%, 20%, and 30%, DKA exhibits higher tensile strength than NP and FA, but it is lower than the control OPC mix (see Figure 24b). As the replacement level of DKA, FA, and NP increases, the tensile strength decreases, as shown in Figure 24. 

### 3.5. Ultrasonic Pulse Velocity (UPV)

**Figure 25:**
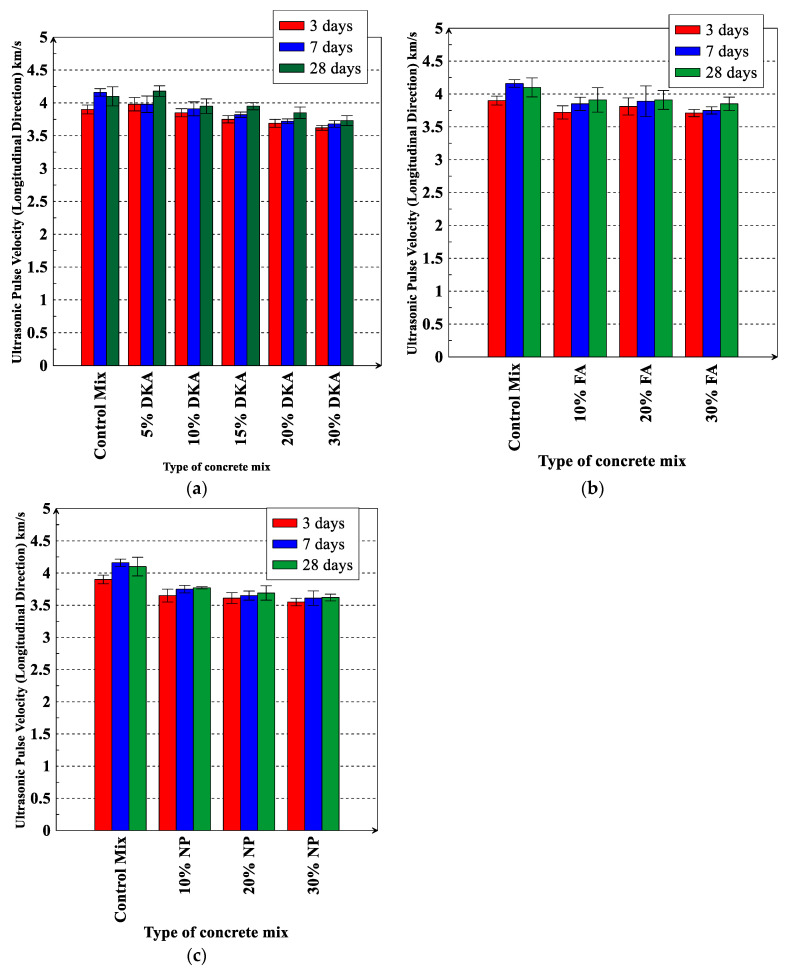
Ultrasonic pulse velocity of concrete mixtures for (**a**) DKA, (**b**) FA, and (**c**) NP. Error bars represent one standard deviation from the average of three specimens.

Figure 25 shows the results of UPV for the various replacement levels of DKA, FA, and NP in the longitudinal direction of the cylindrical concrete samples. The UPV for the control mix was 3.91, 4.16, and 4.1 km/s at 3, 7, and 28 days, respectively. Replacing cement with 5% DKA results in a slight increase in UPV to 3.98, 3.99, and 4.18 at 3, 7, and 28 days, respectively. For the 10%, 20%, and 30% replacement levels, the NP mixes had the lowest UPV when compared to the DKA and FA mixes. It can be noticed in Figure 25 that UPV decreases with an increase in the replacement levels of DKA, FA, and NP from 10% to 30%. The pulse velocity decreases because, at higher replacement levels, the DKA concrete has a less dense matrix with higher porosity. Figure 22 and Figure 25 show a strong correlation between the results of UPV and the compressive strength results. As the UPV increases, the compressive strength increases. The UPV provides a good indication of the quality and strength of concrete mixtures.

## 4. Conclusions

This study investigated the feasibility of using date kernel ash (DKA) as a partial cement replacement material. A comparison was made between DKA, NP, and FA in terms of the evolution of the heat of hydration and thermal and mechanical properties. The following conclusions can be drawn from the experimental results:

- The experimental results show that the calcined date kernel ash has a strong potential for use as a partial replacement of cement in concrete with up to 30% without significantly affecting the hardened properties of concrete. At the 30% DKA replacement level, the 28-day compressive strength is 25.2 MPa, reflecting a decrease of 26.69% compared to the control OPC mix. The DKA concrete outperformed FA and NP concrete in terms of compressive and tensile strengths at ages of 3, 7, and 28 days for the 10%, 20%, and 30% replacement levels.
- DKA was found to generate less heat of hydration compared to NP and FA at 10% and 20% replacement ratios, respectively, while FA generated the least heat of hydration at a 30% replacement of cement. The concrete mix containing 30% DKA had a slower rate of heat and temperature rise when compared with the concrete mixes containing 30% FA or 30% NP.
- The concrete mixes with DKA at 10%, 20%, and 30% replacement have a lower density, lower thermal conductivity, higher thermal resistivity, and higher specific heat compared to the mixes with NP and FA at the same percentages.
- In conclusion, DKA is a promising new partial cement replacement material, and further research is needed to investigate the durability of DKA concrete.

## Figures and Tables

**Table 1:** Chemical analysis of OPC.

Components	SiO_2_	Al_2_O_3_	Fe_2_O_3_	CaO	MgO	SO_3_	LOI
wt.%	19.15	5.09	3.80	61.65	2.40	2.75	2.38

**Table 2:** Physical properties of DKA, FA, and NP.

Properties/Materials	DKA	FA	NP
Specific surface area, cm^2^/g	3850	5580	4400
Specific gravity	1.8	2.2	2.16
Water absorption	4%	1%	1.2%

**Table 3:** Concrete mix proportions.

Material/Mix No.	Cement (kg/m^3^)	DKA (kg/m^3^)	Fly Ash (kg/m^3^)	NP (kg/m^3^)	Water (Liter)	Aggregate			Sand (kg/m^3^)	Admix. PC314 (Liter)
						20 mm (kg/m^3^)	10 mm (kg/m^3^)	5 mm (kg/m^3^)		
Mix 1 (100% OPC) (REF)	480	-	-	-	182.4	550	400	200	560	2
Mix 2 (5%DKA)	456	24	-	-	182.4	550	400	200	560	2
Mix 3 (10% DKA)	432	48	-	-	182.4	550	400	200	560	2
Mix 4 (15% DKA)	408	72	-	-	182.4	550	400	200	560	3
Mix 5 (20% DKA)	384	96	-	-	182.4	550	400	200	560	3
Mix 6 (30% DKA)	336	144	-	-	182.4	550	400	200	560	4
Mix 7 (10% FA)	432	-	48	-	182.4	550	400	200	560	2
Mix 8 (20% FA)	384	-	96	-	182.4	550	400	200	560	2
Mix 9 (30% FA)	336	-	144	-	182.4	550	400	200	560	2
Mix 10 (10% NP)	432	-	-	48	182.4	550	400	200	560	2
Mix 11 (20% NP)	384	-	-	96	182.4	550	400	200	560	2
Mix 12 (30% NP)	336	-	-	144	182.4	550	400	200	560	2

**Table 4:** XRF analysis of DKA, NP, and FA.

Components	wt.%				
	NPKD	DKA 200 °C	DKA 800 °C	NP	FA
Na_2_O	0.0079	0.219	1.77	5.08	0.4
MgO	0.00328	0.946	4.84	3.62	1.75
Al_2_O_3_	0.0649	0.989	1.97	18.9	23
SiO_2_	0.177	3.19	10.1	55.8	52
P_2_O_5_	0.012	1.42	7.31	0.649	0.6
SO_3_	0.038	1.01	8.44	0.36	1.35
Cl	0.0078	0.355	2.08	0.0817	0.25
K_2_O	0.00551	5.82	28.2	2.82	1.56
CaO	0.454	7.21	33.4	11.7	5
Fe_2_O_3_	0.36	0.87	1.52	0.09	11
LOI	98.86	78.8	2.00	0.95	2.3

## Data Availability

The datasets created and/or analyzed during the current study are available upon request from the corresponding author.
